# Reinforcement-Learning-Based Localization of Hippocampus for Alzheimer’s Disease Detection

**DOI:** 10.3390/diagnostics13213292

**Published:** 2023-10-24

**Authors:** Aditya Raj, Golrokh Mirzaei

**Affiliations:** 1 Electrical and Computer Engineering, The Ohio State University, Columbus, OH 43210, USA; raj.74@buckeyemail.osu.edu; 2 Computer Science and Engineering, The Ohio State University, Marion, OH 43302, USA

**Keywords:** reinforcement learning, deep Q-network, hippocampus region localization, Alzheimer’s detection

## Abstract

Alzheimer’s disease (AD) is a progressive neurodegenerative disorder primarily impacting memory and cognitive functions. The hippocampus serves as a key biomarker associated with AD. In this study, we present an end-to-end automated approach for AD detection by introducing a reinforcement-learning-based technique to localize the hippocampus within structural MRI images. Subsequently, this localized hippocampus serves as input for a deep convolutional neural network for AD classification. We model the agent–environment interaction using a Deep Q-Network (DQN), encompassing both a convolutional Target Net and Policy Net. Furthermore, we introduce an integrated loss function that combines cross-entropy and contrastive loss to effectively train the classifier model. Our approach leverages a single optimal slice extracted from each subject’s 3D sMRI, thereby reducing computational complexity while maintaining performance comparable to volumetric data analysis methods. To evaluate the effectiveness of our proposed localization and classification framework, we compare its performance to the results achieved by supervised models directly trained on ground truth hippocampal regions as input. The proposed approach demonstrates promising performance in terms of classification accuracy, F1-score, precision, and recall. It achieves an F1-score within an error margin of 3.7% and 1.1% and an accuracy within an error margin of 6.6% and 1.6% when compared to the supervised models trained directly on ground truth masks, all while achieving the highest recall score.

## 1. Introduction

Alzheimer’s disease (AD) is a neurodegenerative disorder and one of the leading causes of dementia. To effectively detect and clinically diagnose AD, it is crucial to develop precise and efficient classification tools based on advanced neuroimaging techniques. Structural magnetic resonance imaging (sMRI) is a widely used neuroimaging method for diagnosing Alzheimer’s [1,2]. sMRI can be used as a non-invasive imaging tool to capture atrophies in brain structure and study its progression over time to determine the risk of AD. These studies have consistently shown that the hippocampus is significantly affected in AD [3,4]. Additionally, researchers have identified the texture of the hippocampal region as a valid biomarker, in addition to the hippocampal volume [5,6,7]. However, utilizing the hippocampus for AD detection necessitates the accurate extraction of this region from 3D sMRIs.

While structural MRI (sMRI) offers numerous advantages, it does come with certain limitations. One of these limitations is that structural brain changes can occur in the later stages of the disease, making early detection challenging. Additionally, the quality of sMRI imaging can be poor, rendering traditional image processing tools less effective for feature extraction. In recent years, with advancements in image processing techniques, machine learning algorithms have become more efficient at extracting features from noisy sMRI data. Consequently, various deep learning methods have been employed to extract features from these noisy image modalities for the purpose of disease detection [8,9,10,11,12,13]. Depending on the feature extraction process, these detection methods can be categorized into 2D-based and 3D-based techniques. Two-dimensional-level feature extraction offers a reduced computational cost, but it may result in learning a less comprehensive feature representation. Conversely, 2D-based methods often underutilize the information present in the 3D sample. In 3D-based feature learning, popular approaches include the use of 3D convolutional neural-network-based deep learning models with the input either being the entire 3D sMRI sample or a patch from it. This approach can learn global features but comes at the cost of higher computational requirements.

Several machine learning algorithms have been applied to study the hippocampus region for AD detection [14,15,16,17]. Shen et al. [18] investigated hippocampal shape variations using statistical shape models and performed AD vs. control (CN) classification using support vector machines. Another approach involves segmenting the hippocampus, which can serve as a powerful detection technique. Several algorithms have been introduced for hippocampus volume segmentation [19,20], which is subsequently employed for AD classification. Liu et al. [20] proposed a joint hippocampus segmentation and AD classification method using a 3D Dense Net as the backbone, achieving an accuracy of 88.9% in binary AD vs. CN classification. However, the majority of these algorithms suffer from high computational costs due to processing volumetric data. Additionally, various region proposal and object detection techniques have been developed [21,22] to focus on extracting relevant regions of interest. These methods can be used to design more efficient classifiers with reduced computational requirements. The localization of the hippocampal region compared to the segmentation of its volume has been shown to have a similar sensitivity for AD [23,24]. Many of these approaches employ deep convolutional neural networks with voxel-based feature extraction techniques [9,25]. While a voxel-based approach offers the advantage of learning from global features, it comes with excessive computational overhead, given that sMRIs are high-dimensional 3D scans. Moreover, the detection of AD from texture has been much less explored than using the entire volumetric information. Further, ref. [26] conducted a review of region-of-interest-based methods that utilize a 3D CNN for extracting hippocampal features and classifying Alzheimer’s disease (AD) from sMRI data. They observed that the hippocampus and its surrounding region can play an important role toward Alzheimer’s detection from sMRIs. Therefore, the localization of the hippocampus and its surrounding region, without the explicit segmentation of the hippocampus, can offer valuable insights. Additionally, localized hippocampal regions can be extracted using less computationally intensive methods. Consequently, it is imperative to explore localization as an important approach in addition to traditional segmentation techniques.

In this work, we propose a novel reinforcement-learning-based algorithm for localizing the hippocampal region from structural MRIs and implement a classifier that relies solely on the region of interest (hippocampal region) as the 2D input. Our method leverages a deep Q-network (DQN) and convolutional neural network (CNN) within a classification framework, employing a single optimal slice extracted from each subject’s 3D sMRI. This approach significantly reduces computational complexity while maintaining a performance comparable to volumetric data analysis methods. To evaluate the model’s performance, we compare it with other 2D CNN-based supervised models trained on ground truth hippocampal masks. Furthermore, we employ an integrated loss function consisting of cross-entropy and contrastive loss to train the classifier model. The followings are the main contributions of this work:

- Introduction of a novel reinforcement-learning-based algorithm for localizing the hippocampal region in structural MRIs.
- Application of an integrated loss function combining cross-entropy and contrastive loss to effectively train the classifier model.
- Utilization of a deep Q-network (DQN) and convolutional neural network (CNN) framework for classification, which involves the use of a single optimal slice extracted from each subject’s 3D sMRI, thereby reducing the complexity while still providing comparable results.
- Comparison of the model’s performance with that of other 2D CNN-based supervised models trained on ground truth hippocampal masks.

The remainder of the paper is organized as follows: the methodology section covers details of the dataset used and preprocessing steps, introduces the proposed model, and provides background information on deep Q-learning. Subsequently, the experimental setup section presents details of the reinforcement learning approach, explaining designed actions, rewards, and the training of the Policy and Target Net. Finally, the results and discussion section delves into the performance metrics for the proposed algorithm, followed by a comparison with existing methods and an ablation study.

## 2. Methodology

### 2.1. Dataset and Preprocessing

The data used in this study were obtained from Alzheimer’s Disease Neuroimaging Initiative (ADNI, http://adni.loni.usc.edu/ (accessed on 5 January 2021)). We conducted a subject-level data division, separating subjects into training and testing sets in such a way that no sample seen during training would be repeated during testing. The dataset used was ADNI-1 with a total of 255 subjects. There were 100 samples for AD and 100 for CN. We performed 5-fold cross-validation on a held-out test set, comprising 55 samples (25 samples for CN and 30 samples for AD). Additionally, we extracted bounding boxes for the hippocampus mask provided for each sample of 3D sMRI. Due to variations in the number of slices and image dimension across different samples, we resized each sample to have dimensions of depth×256×256. In this work, we subdivided the dataset into two subsets: 2D slice set and 3D set. We trained the proposed model and present results on reinforcement learning (RL)-based localization technique for both sets.

**Figure 1:**
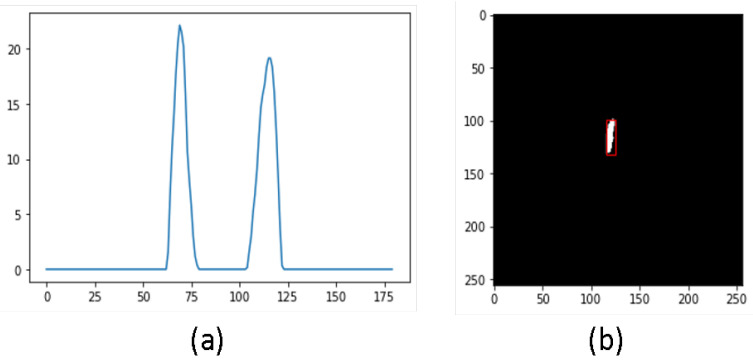
(**a**) Mean mask values per slice and (**b**) mask with maximum mean.

Two-dimensional slice set—For this subset, we selected the optimal slice from a given sequence of masks, ensuring one slice per sample. Figure 1a shows the mean values of masks for all slices within a sample. It should be noted that most slices lack masks. Therefore, we extracted the slice with the highest mean value of mask pixel intensities (seen as peaks in the figure), as exemplified in Figure 1b. To extract the hippocampus bounding box, we employed Otsu thresholding method on the masks. Subsequently, we analyzed contours around the thresholded masks, with the contour possessing the largest area providing the bounding box dimensions. The image slice with the highest mean value corresponding to the mask was selected as the input, while the remaining slices were discarded. Finally, we applied min-max normalization across all samples.

3D slice set—In this set, we preprocessed samples to have image dimensions of depth×256×256. We selected slices with the most informative content across all samples, resulting in a uniform depth of 145 slices per sample. This reshaping standardized the samples to dimensions of 145×256×256. We followed the same protocol as described above to extract hippocampus for each slice within a sample. Finally, min-max normalization was applied across all samples. The train and test set split was kept consistent with the 2D slice set.

### 2.2. Proposed Method

In this study, we introduce a reinforcement-learning-based algorithm using Q-learning designed for the localization of hippocampal region in structural MRIs.

Q-Learning [27] is a popular reinforcement learning algorithm that can be used to learn policy from a model-free setup. Q-learning does not need prior knowledge of the reward function or the dynamics of the environment. It works by updating the action-value function or Q-values for every action that agent takes. These Q-values are stored in a table, where the number of rows corresponds to the number of states and the number of columns is the same as the total number of actions that the agent can take. The Q-values are updated using temporal difference method in RL. Suppose for a given action *a* and current state *s* that the action value function can be denoted as Q(a,s). Further, the Bellman equation can be employed to update Q, say after the agent takes an action $a^{\prime }$ to transit into a new state $s^{\prime }$, as:

(1) $$Q\left(s,a\right)=Q\left(s,a\right)+\alpha (r+\gamma \;max\left(Q\left(s^{\prime },a^{\prime }\right)\right)-Q\left(s,a\right))$$

where α is the step length used to update the value of Q(a,s) and γ is the discontinuing factor with a value in the range {0,1}. It is used to discount future rewards because more importance is placed on the current reward. Some of the limitations of Q-learning include the requirement of large exploration examples for updating the Q-table, which makes Q-learning slow to converge.

We propose an algorithm based on deep Q-network (DQN) featuring 2D and 3D convolutional neural networks (CNNs) for policy learning. For the 2D experiments, we employ the DQN [28] and a 2D CNN-based framework for classification task. This framework operates on a single optimal slice extracted from each subject’s 3D sMRI. Additionally, within the realm of 2D-based experiments, we introduce two variants. One variant exclusively processes the extracted hippocampal region, while the other employs a fusion network. The fusion network takes both extracted features of hippocampal region and the corresponding sample as input, producing a feature vector as its output. This fused feature representation is then used as input for the final prediction network. In contrast, for the 3D model, we reshape the samples and directly localize the 3D hippocampal region from 3D sMRI samples. Our results demonstrate that a 3D model performs well on the task of hippocampus localization and downstream task of AD vs. CN classification. However, the 2D-based model outperforms the proposed 3D model. Consequently, we focus on comparing our model with the 2D model only. We evaluate the performance of the 2D model by comparing it to other 2D CNN-based supervised models trained on ground truth hippocampal masks. The comparison establishes a performance ceiling for the AD vs. CN detection task.

**Figure 2:**
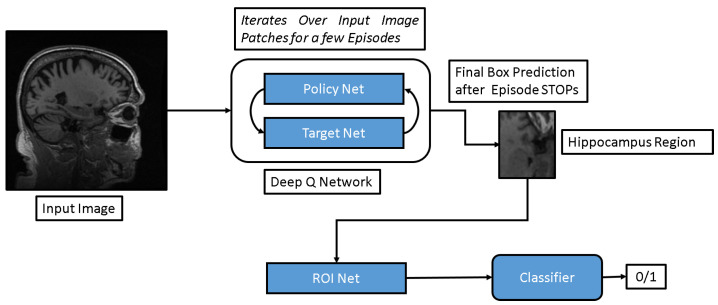
Framework of ROI Net followed by CNN-based classifier.

The inputs to the DQN model comprise 2D slices categorized as either AD or CN, all of which have undergone preprocessing. This DQN network comprises two key components: Policy Net and Target Net. Both networks are structured with three convolution layers followed by two fully connected layers. During training, the Policy Net is updated after each episode, while the Target Net shares weights with the Policy Net every few episodes and is not trained separately. During testing, only the Target Net is utilized for predictions. Subsequently, the output of the DQN model feeds into the ROI Net, as depicted in Figure 2. The ROI Net consists of three 2D convolutional layers, followed by two fully connected layers for classification. The ROI Net’s output provides a prediction score of 1 for AD and 0 for CN.

The primary objective of the DQN model is to localize the hippocampal region within the input image. This task can be formulated as a Markov decision process (MDP), where the input image functions as the environment, and the agent interacts with this environment by focusing on a small bounding box region and executing a predefined set of actions. The agent’s goal is to identify a bounding box containing the hippocampus region. The agent terminates its search once it successfully identifies such a bounding box. The agent has a state representation that corresponds to the region that it is currently focusing on, and based on its actions, it receives reward. The expected outcome is that the agent learns a generalizable policy, allowing it to execute the correct sequence of actions, starting from any given region in the input image, ultimately achieving its goal of locating the hippocampal region.

**Figure 3:**
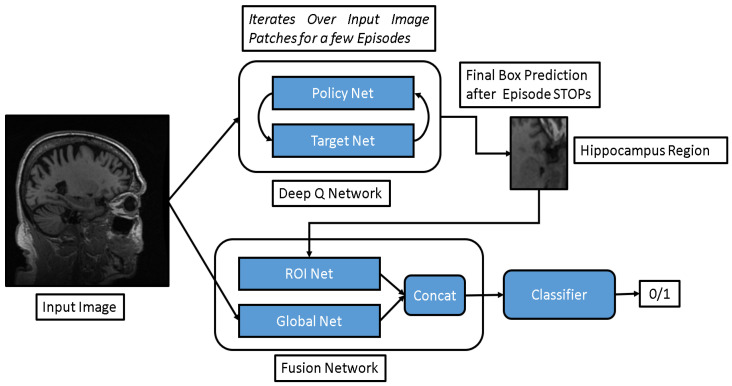
Framework of Fusion Net (including ROI and Global feature extraction) followed by CNN-based classifier.

In the CNN model with Fusion Net (F(G,R) (see Figure 3)), the input to the Fusion Net is formed by combining the output of two distinct models: the ROI Net (R) and the Global Net (G). The Global Net is responsible for extracting features from the entire 2D slice, while the ROI Net extracts features specifically from the localized hippocampal region. The outputs of these two networks are then combined and fed into the Fusion Net to learn a joint feature representation. Subsequently, this fused feature vector is utilized for the AD vs. CN classification task. For the 3D sample-based DQN-CNN algorithm, we adapt the ROI Net and Fusion Net to incorporate 3D convolutional layers instead of 2D. The model structure remains consistent with what is depicted in Figure 2 and Figure 3. A detailed description of the 3D ROI Net is given in Table 1. The input dimension for the first layer of the model is 50 for ROI Net and 145 for Global Net. Similarly, the output dimension is set to 512 for ROI Net and 1024 for Global Net. For the 2D model, the layer order remains the same but 3D convolutions are replaced with 2D convolutions.

## 3. Experimental Setup

### 3.1. Designed Actions

**Figure 4:**
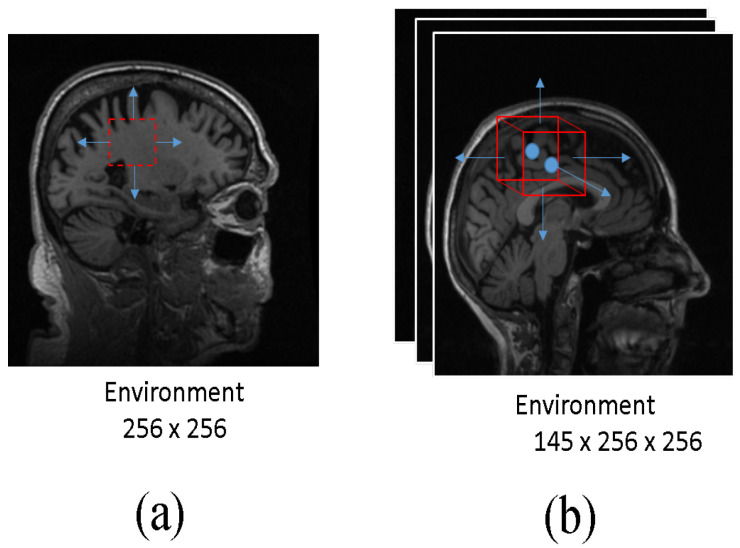
Experimental setup with agent shown as the red box and arrows depicting the permissible actions for (**a**) 2D data and (**b**) 3D data. In the case of 2D data, there are five actions available (Up, Down, Left, Right, and Terminate), while for 3D data, there are two additional actions, including Top and Bottom.

In our RL-based approach, we have defined a set of five actions: Up, Down, Left, Right, and Terminate (refer to Figure 4). The action Up shifts the bounding box in the image vertically by 5 positions, while Down moves it downward by 5 units along the *y*-axis. Similarly, Left and Right actions result in the bounding box moving horizontally by 5 units to the left and right along the *x*-axis, respectively. The Terminate action ends the episode and the DQN model generates the bounding box that encompasses the desired region of interest. For our 3D-based localization, we have introduced two additional actions: Top and Bottom. When Top is selected, the bounding box moves upward along the depth (*z*-axis) of the sample by 5 units. Conversely, the Bottom action causes the bounding box to descend in depth along the *z*-axis by 5 units.

### 3.2. Reward Computation

We utilize an ϵ-greedy algorithm for training the RL agent. During the initial 10 episodes, the agent selects actions randomly. Subsequently, the value of ϵ remains fixed at 0.2. Each action results in the generation of a new bounding box, and we compute the reward ($R_{a}$) for action *a* using the following formulation (as shown in Equation (3)), which aligns with the reward function employed in [21].

(2) $$\begin{array}{c} \mathrm{IoU}(\mathrm{bb},\mathrm{gt})\;=\;area(\mathrm{bb}\cap \mathrm{gt})/area(\mathrm{bb}\cup \mathrm{gt}) \end{array}$$

(3) $$\begin{array}{c} R_{a}\left(s,s^{\prime }\right)=sign\left({\mathrm{IoU}(\mathrm{bb}}^{\prime },\mathrm{gt})-\mathrm{IoU}(\mathrm{bb},\mathrm{gt})\right) \end{array}$$

where bb′ represents the new bounding box, gt is the ground truth for bounding box, *s* is the the previous state, $s^{\prime }$ is the new state, and bb is the previous bounding box. Essentially, this reward function assigns a positive reward of 1 when the agent’s action brings it closer to the ground truth bounding box; otherwise, a reward of −1 is assigned. Subsequently, the action values learned through the policy network are updated based on the computed reward.

### 3.3. Deep Q-Network Setup

The initial position of the bounding box (x,y) is set randomly. This bounding box has fixed dimensions, with a width of 50 and a height of 65. These dimensions were chosen to accommodate the maximum possible size of the hippocampus.

For each action chosen, the agent is allowed to move a fixed number of steps, equivalent to 5 units in the selected direction. In addition, if, during an episode, the agent encounters the boundaries of the environment, the bounding box is reset to the position (x,y) of (100,100) for the 2D model or (x,y,z) to the position of (100,100,50) for the 3D model, without restarting the episode. The episode concludes when either the agent reaches its goal or the predefined limit for the total number of episodes is reached, which has been set to 100.

### 3.4. Model Training Protocol

Upon the conclusion of each episode, a bounding box is predicted to encompass the hippocampus region. This region is then cropped from the image and resized to 256×256 pixels. Subsequently, it is input into the CNN-based classifier model, which produces a binary label: 1 for AD and 0 for CN. The proposed DQN model undergoes training for 10 epochs, and the total number of episodes is capped at 100. We utilize *Adam* optimizer with a learning rate of 0.01 and employ the *Smooth L1 Loss* function. For training the ROI Net, we employ binary cross-entropy loss (C1) along with a modified contrastive loss (C2). The following formulation (see Equation (6)) shows the final loss function:

(4) $$\begin{array}{c} C1\;=\;\frac{1}{N}\Sigma_{i}y_{i}\log\left({\hat{y}}_{i}\right)+\left(1-y_{i}\right)\log\left(1-{\hat{y}}_{i}\right) \end{array}$$

(5) $$\begin{array}{c} C2\;=\;\frac{1}{N}\Sigma_{i}\left(1-y_{i}\right)\left({\hat{y}}_{i}^{2}\right)+m*y_{i}{\left(max\left(1-{\hat{y}}_{i},0\right)\right)}^{2} \end{array}$$

(6) $$\begin{array}{c} Loss=C1+2*C2 \end{array}$$

where $y_{i}$ is the true label, ${\hat{y}}_{i}$ is the predicted label, and *m* is a hyperparameter set to 2. Finally, the classifier network is trained for 60 epochs with *Adam* optimizer and learning rate of $9\times 10^{-5}$.

### 3.5. Evaluation Metric

To evaluate the classification performance of the proposed model we employ the following measures: accuracy, F1-score, precision, recall, and balanced accuracy. To measure these metrics, we use true positive (*TP*), true negative (*TN*), false positive (*FP*), and false negative (*FN*). Here, *TP* denotes the positive class predicted as positive by the classifier, *TN* denotes the negative class predicted as negative, *FP* denotes the negative class predicted as positive, and *FN* denotes the positive class predicted as negative.

(7) $$\begin{array}{c} \mathrm{Accuracy}=\frac{TP+TN}{TP+TN+FP+FN} \end{array}$$

(8) $$\begin{array}{c} \mathrm{Precision}=\frac{TP}{TP+FP} \end{array}$$

(9) $$\begin{array}{c} \mathrm{Recall}=\frac{TP}{TP+FN} \end{array}$$

(10) $$\begin{array}{c} F1-\mathrm{score}=\frac{2\times Precision\times Recall}{Precision+Recall} \end{array}$$

Additionally, we compute the balanced accuracy (BA) instead of AUC for comparison with existing methods. Formula for the balanced accuracy can be given as follows:

(11) $$\begin{array}{c} \mathrm{Sensitivity}=\frac{TP}{TP+FN} \end{array}$$

(12) $$\begin{array}{c} \mathrm{Specificity}=\frac{TN}{TN+FP} \end{array}$$

(13) $$\begin{array}{c} \mathrm{Balanced}\;\mathrm{Accuracy}=\frac{Sensitivity+specificity}{2} \end{array}$$

## 4. Results

We conducted hippocampal region localization using DQN, where the agent’s objective was to extract a patch of dimensions 50×65 from an input image of size 256×256 in the case of the 2D model, and a patch of 50×65×50 from an input of size 145×256×256 for the 3D model. The extracted patch must contain the hippocampal region for the agent to achieve its goal. The 2D trained model demonstrated a satisfactory localization performance, achieving success over ≈90% of cases. During testing, the DQN model successfully extracted usable patches in 50 out of 55 held-out samples.

In contrast, in the experiments based on the 3D model, the model’s performance falls short compared to the 2D-model-based localization. The 3D model successfully extracted a satisfactory region of interest in only 50–60% of cases. One possible explanation for this discrepancy can be attributed to the increased complexity stemming from the larger number of states and actions for the RL agent in the 3D model when compared to the 2D model.

**Figure 5:**
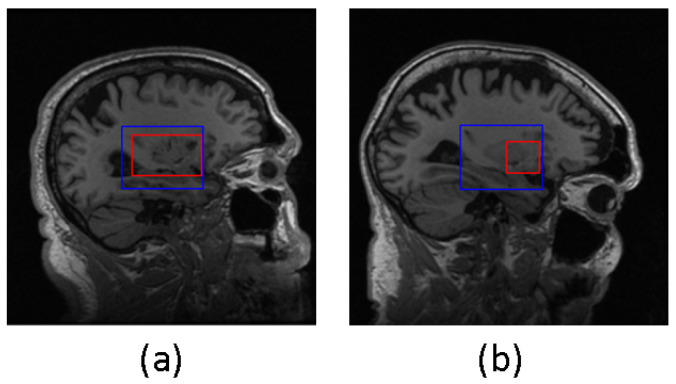
Satisfactory localization outcomes from DQN in both examples (**a**) and (**b**). Blue box represents the predicted bounding box, and red box represents the truth hippacapus region.

**Figure 6:**
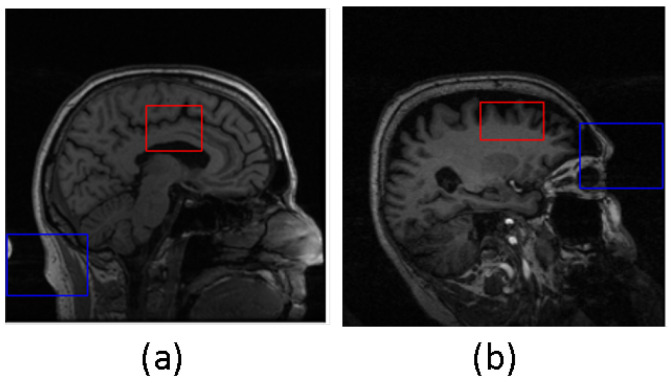
Poor localization outcomes from DQN. Blue box represents the predicted bounding box, and red box represents the truth hippacapus region. In (**a**) the hippocampal region was correctly extracted but the agent failed to generate the bounding box that properly encloses the hippocampal region. In (**b**) the error lies in the accurate representation of the hippocampal ground truth itself.

The samples in which the proposed algorithms perform poorly were not excluded from the final classification results. In Figure 5, two examples are presented where the predicted bounding box (shown in blue) accurately encompasses the ground truth hippocampal region (shown in red). In contrast, Figure 6 shows two instances where the agent fails to produce an acceptable bounding box. This issue can be attributed to two factors: firstly, as observed in the example shown in Figure 6a, the hippocampal region was correctly extracted but the agent failed to generate the bounding box that properly encloses the hippocampal region. Secondly, as seen in Figure 6b, the error lies in the accurate representation of the hippocampal ground truth itself.

Following the output of the DQN, we utilized the ROI Net and Fusion Net algorithms to obtain the final prediction score. To assess the classifier’s performance, we trained the same 2D CNN model used in the classifier but with ground truth hippocampus masks as input. Additionally, we employed AlexNet as a feature extractor followed by fully connected layers for class prediction. The results were averaged over five-fold cross validation, where test scores were computed on a held-out set of 55 samples. Table 2, shows the results of 2D models ROI Net and Fusion Net. The DQN-CNN method with ROI Net achieved an F1-score of 69.2%, which was comparable to the scores of supervised models trained directly on ground truth masks. Notably, the performance of AlexNet was 3.7% and 17.4% higher than the proposed DQN-CNN with the ROI Net method for F1-score and precision, respectively. Similarly, the 2D CNN achieved 1.1% and 3.6% higher scores for F1-score and precision when compared to ROI Net. However, the proposed model with Fusion Net achieved the best recall, with an improvement of 9.8% and 5.2% over AlexNet and the 2D CNN, respectively. Additionally, the proposed Fusion Net outperformed other methods overall, achieving an F1-score of 75%. Additional experiments with 3D ROI Net yielded an inferior performance in the task of AD vs. CN classification. This can be directly attributed to the poor localization results. Moreover, adding additional permissible actions and increasing the state space of the environment significantly increased the complexity. Consequently, the model then required a substantial increase in exploration experience, leading to considerable longer training times. For instance, the average training time for the 3D ROI Net was ≈200 h, whereas the average training time for the 2D ROI Net was ≈9 h. Table 2 shows the accuracy achieved by various computational methods in the classification task. The 2D CNN and AlexNet, both trained directly on hippocampus ground truth masks, achieved accuracies of 71.6% and 76.67%, respectively. This can serve as a benchmark for comparing the proposed methods. The 2D DQN-based ROI Net, trained exclusively using the extracted hippocampal region, achieved an accuracy of 70%, whereas its 3D extension had the lowest accuracy, at 60%. In the case of the 2D Fusion Net, where both input slice and the extracted hippocampal region were used as input to the classifier model, it demonstrated a performance that was comparable to the pre-trained AlexNet model trained on the ground truth mask.

## 5. Discussion

### 5.1. Comparative Analysis of Existing Hippocampus Localization Methods

Table 3 presents a performance comparison among various methods for the AD vs. CN classification task, focusing on the information derived from the hippocampus. Most of these methods utilize hippocampus volumes (HVs) as input to classifier models. For instance, Katabathula [29] conducted experiments using shape features extracted from within the hippocampus, employing the LB spectrum technique, and achieved an accuracy rate of 70.89%. In our study, we took a different approach by utilizing a single slice from structural MRI data to extract the entire hippocampus within the HVs. In contrast to methods that rely on texture or shape features, our RL-based localization and classification method outperforms them, demonstrating a comparable and even superior accuracy rate of (76.67%) in the AD vs. CN classification task.

### 5.2. Ablation Study: Episode Termination

**Figure 7:**
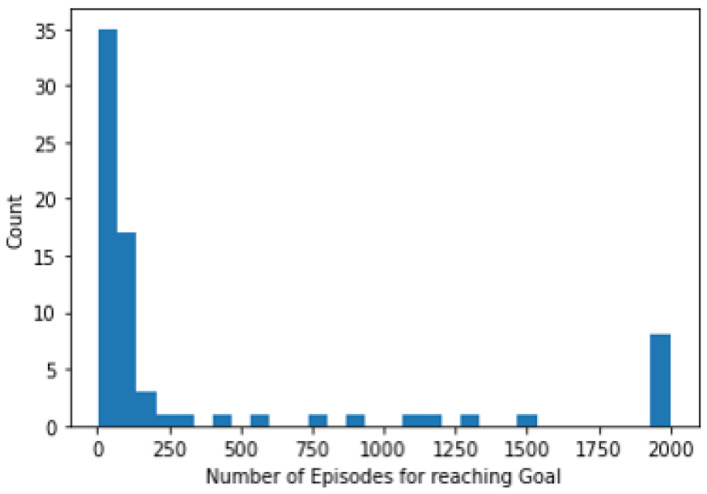
Histogram depicting the number of episodes required to reach the region of interest.

We conducted additional experiments on the DQN ROI Net (2D model) to investigate two strategies for terminating the episodes within the RL algorithm. In the first approach, the agent selects the Terminate action, resulting in an immediate end to the episode. Subsequently, the model provides the last region accessed in the image as the localization result. In the second approach, the agent is allowed to take no action when the Terminate action is selected. In this scenario, the episode continues for a fixed duration of 100 episodes before concluding, with the localization output being the last region accessed by the agent.The results shown in Figure 3 were obtained using this second strategy. In contrast, when employing the first strategy, the RL agent is allowed to exit the episode as soon as the Terminate action is chosen. This offers the advantage of designing a faster localization algorithm. However, it adversely impacts the overall performance of the model on the classification task. As depicted in Figure 7, we present a plot illustrating the relationship between the count and the number of episodes required for the agent (in the case of the 2D model) to converge to the goal in the first scenario mentioned above. Notably, approximately ≈85% of the time, the agent completes its goal within ≤200 episodes. However, the accuracy achieved by this model on AD vs. CN classification using the DQN ROI Net was 62%, which is 8% lower than the accuracy achieved by employing the second strategy.

## 6. Conclusions

In this paper, we introduce a novel reinforcement-learning-based localization and a convolutional-neural-network-based classification algorithm. Our approach, termed DQN-CNN, is designed to enable an RL agent to learn an optimal policy for localizing the hippocampus region and subsequently performing classification on the extracted patch. To guide the agent effectively, we employ reward-shaping techniques to ensure its accurate positioning on the patch containing the hippocampal region. This localized patch is then utilized for the Alzheimer’s disease (AD) vs. cognitive normal (CN) classification task. Furthermore, we introduce an integrated loss function that combines cross-entropy and contrastive loss to effectively train the classifier model. We train supervised learning frameworks on ground truth hippocampus patches to establish the highest possible classification performance for our proposed model.

Our technique offers an automated end-to-end approach for hippocampus localization and classification, significantly reducing computational complexity while yielding promising results that are comparable to techniques relying on ground truth hippocampus masks. Our results emphasize the efficacy of our approach in leveraging 3D sMRI data for hippocampal extraction and classification, potentially offering a promising avenue for improved AD diagnosis.

## Figures and Tables

**Table 1:** Detailed architecture of 3D ROI Net. This architecture was kept the same for 3D Target Net/Policy Net and 3D Global Net.

3D ROI Net (Same as 3D Target Net)	
Conv 3D + LeakyReLU	(Input dim, 32), filter size = 5, stride = 2
Maxpool 3D	(2, 2, 2)
Conv 3D + LeakyReLU	(32, 64), filter size = 5, stride = 1
Maxpool 3D	(1, 2, 2)
Conv 3D + LeakyReLU	(64, 64), filter size = 3, stride = 2
Maxpool 3D	(2, 2, 2)
Flatten Features	
Dense + ReLU	(Input dim, 256)
Dense + Sigmoid	(256, Output dim)

**Table 2:** Comparison of performance metrics for classification of AD vs. CN in 2D model (HR: the method through which the Hippocampus Region is accessed).

Tech.	Accuracy	F1-Score	Recall	Precision	HR
2D CNN	71.6%	70.3%	64.8%	77.6%	Ground Truth
AlexNet	76.6%	72.9%	61.2%	91.4%	Ground Truth
Proposed ROI Net	70%	69.2%	65%	74%	DQN
Proposed Fusion Net	76.67%	75%	70%	83%	DQN

**Table 3:** Comparison of the proposed framework with existing methods for AD vs. CN classification with hippocampus localization (HV: hippocampus volume, H: hippocampus, ROI: region of interest, DL: deep learning, L: left, R: right). Balanced accuracy * is the average of sensitivity and specificity.

	Tech.	Data	Accuracy	F1-Score	Balanced Accuracy *
	KNN	HV	85.52%	76.59%	82.07%
[30]	RF	HV	86.84%	79.16%	83.5%
	SVM	HV	88.15%	79.06%	85.47%
[29]	DL	Shape	70.89%	63.14%	64.86%
		Shape + Vis	92.52%	91.45%	91.32%
[31]	DL	L. HV ROI	80.40%	85.16%	80.46%
		R. HV ROI	79.5%	79.1%	79.39%
[32]	DL	HV Mask	-	-	**76.6**%
		HV Texture	-	-	**78.8**%
	RL	2D H ROI	70%	69.2%	69.5%
Ours	+DL	2D H ROI +	76.67%	75%	76.5%
		Whole slice			

## Data Availability

The code underlying this article are available in Github and can be accessed with https://github.com/AR13ar/DQN_based_Localization (accessed on 13 October 2023).

## Abbreviations

The following abbreviations are used in this manuscript:

RL	Reinforcement Learning
DL	Deep Learning
SVM	Support Vector Machine
KNN	K-Nearest Neighbor
ROI	Region of Interest
AD	Alzheimer’s Disease
MCI	Mild Cognition Impairment
CN	Cognitively Normal
HV	Hippocampus Volume
BA	Balanced Accuracy
